# The bovine nasal fungal community and associations with bovine respiratory disease

**DOI:** 10.3389/fvets.2023.1165994

**Published:** 2023-06-27

**Authors:** Ruth Eunice Centeno-Martinez, Suraj Mohan, Josiah Levi Davidson, Jon P. Schoonmaker, Aaron Ault, Mohit S. Verma, Timothy A. Johnson

**Affiliations:** ^1^Department of Animal Science, Purdue University, West Lafayette, IN, United States; ^2^Department of Agricultural and Biological Engineering, Purdue University, West Lafayette, IN, United States; ^3^Department of Electrical and Computer Engineering, Purdue University, West Lafayette, IN, United States; ^4^Weldon School of Biomedical Engineering, Purdue University, West Lafayette, IN, United States; ^5^Birck Nanotechnology Center, Purdue University, West Lafayette, IN, United States

**Keywords:** bovine respiratory disease, ITS gene, qPCR, cattle, nasal mycobiome

## Abstract

**Introduction:**

Effective identification and treatment of bovine respiratory disease (BRD) is an ongoing health and economic issue for the dairy and beef cattle industries. Bacteria pathogens *Pasteurellamultocida*, *Mycoplasmabovis*, *Mannheimia haemolytica*, and Histophilus somni and the virus Bovine herpesvirus-1 (BHV-1), Bovine parainfluenza-3 virus (BPIV-3), Bovine respiratory syncytial virus (BRSV), Bovine adenovirus 3 (BAdV3), bovine coronavirus (BoCV) and Bovine viral diarrhea virus (BVDV) have commonly been identified in BRD cattle; however, no studies have investigated the fungal community and how it may also relate to BRD.

**Methods:**

The objective of this study was to understand if the nasal mycobiome differs between a BRD-affected (n = 56) and visually healthy (n = 73) Holstein steers. Fungal nasal community was determined by using Internal Transcribed Spacer (ITS) sequencing.

**Results:**

The phyla, Ascomycota and Basidiomycota, and the genera, *Trichosporon* and *Issatchenkia*, were the most abundant among all animals, regardless of health status. We identified differences between healthy and BRD animals in abundance of *Trichosporon* and *Issatchenkia orientalis* at a sub-species level that could be a potential indicator of BRD. No differences were observed in the nasal fungal alpha and beta diversity between BRD and healthy animals. However, the fungal community structure was affected based on season, specifically when comparing samples collected in the summer to the winter season. We then performed a random forest model, based on the fungal community and abundance of the BRD-pathobionts (qPCR data generated from a previous study using the same animals), to classify healthy and BRD animals and determine the agreement with visual diagnosis. Classification of BRD or healthy animals using ITS sequencing was low and agreed with the visual diagnosis with an accuracy of 51.9%. A portion of the ITS-predicted BRD animals were not predicted based on the abundance of BRD pathobionts. Lastly, fungal and bacterial co-occurrence were more common in BRD animals than healthy animals.

**Discussion:**

The results from this novel study provide a baseline understanding of the fungal diversity and composition in the nasal cavity of BRD and healthy animals, upon which future interaction studies, including other nasal microbiome members to further understand and accurately diagnose BRD, can be designed.

## Introduction

Bovine respiratory disease (BRD) is a respiratory disease in which microbes infect the lung of dairy and beef cattle lungs causing morbidity and mortality (1, 2). Animal death, reduction of feed efficiency, and BRD treatment are estimated to cost the industry $800–$900 M per year in the United States (3). One study reported that producers lose $40.46/calf for one BRD treatment, $58.35/calf for two treatments, and $291.93/calf for three or more treatments (4), demonstrating the importance of controlling the spread of the disease within the herd. Additionally, it has been shown that BRD develops by the action of multiple factors including environmental (e.g., ambient temperature, humidity, ventilation), predisposing (animal age, stress, transportation), and epidemiological agents, such as bacteria (*P. multocida, H. somni, M. haemolytica* and *M. bovis*), viruses bovine herpesvirus-1 (BHV-1), bovine parainfluenza-3 virus (BPIV-3), bovine respiratory syncytial virus (BRSV), bovine adenovirus 3 (BAdV3), bovine coronavirus (BoCV) and bovine viral diarrhea virus (BVDV) (5–7).

In the cattle industry, detecting respiratory disease in cattle is often based on the observing animal behavior (such as depression, appetite loss) and appearance (such as nasal discharge, increase respiratory rate, moist cough, temperature elevation) (8). Different scoring system have been develop to identify BRD such as the DART method that uses depression, appetite loss, respiratory character change and rectal temperature (8) as clinical signs of disease. However, this visual-based method has a low sensitivity (62%) and specificity (63%) in differentiating healthy and BRD animals because visual symptoms are not unique to respiratory disease (9–11). Thus, it is necessary to identify a method that will improve BRD detection in cattle. Quantifying the abundance of BRD-pathogens present in the nasal cavity or nasopharynx might help discriminate BRD from healthy animals (2, 12–15). As an example, we previously quantified the abundance of the BRD-pathobionts *M. haemolytica, P. multocida, H. somni*, and *M. bovis* in the nasal cavity of healthy and BRD animals (12). We showed that the abundance of *H. somni, M. haemolytica,* and *M. bovis* were significantly higher in BRD than healthy animals. In addition, we performed a random forest analysis using the nasal bacterial microbiome predicted by 16S rRNA gene sequencing and the quantification of BRD-pathobionts to classify an animal as healthy or BRD. A total of 11 out of 20 cattle visually identified as BRD animals, and 12 out of 30 clinically healthy animals agreed with the visual diagnosis and the classification predicted by the nasal community (16S rRNA, model accuracy of 68%) and BRD-pathobiont abundance (model accuracy of 66%). These results indicate the potential of utilizing the nasal microbiome composition to classify healthy and BRD animals.

As the knowledge regarding bacteria associated with BRD increases, it is important to also analyze the role of viruses and fungi. Viral infections of bovine respiratory disease have been related to the presence of Bovine herpesvirus-1 (BHV-1), Bovine parainfluenza-3 virus (BPIV-3), Bovine respiratory syncytial virus (BRSV), Bovine adenovirus 3 (BAdV3), bovine coronavirus (BoCV) and Bovine viral diarrhea virus (BVDV) (16–20). These viruses are hypothesized to damage respiratory and lung parenchyma, which can facilitate the translocation of bacterial pathogens and cause a delay in the animal immune response to bacterial infection (21, 22). On the other hand, the presence of fungal pathogens has been identified in cattle (23). As an example, the presence of *Candida* species have been related with mastitis, abortion, otitis extrema, gastrointestinal infection, disseminated candidiasis, bronchopneumonia in cattle (23). In addition, fungi from the *Mucorales* order have been associated with murcormycotic ruminitis and lymphadenitis in cattle and the fungi *Aspergillus fumigatus* caused bovine mycotic placentitis and abortion (24, 25).

In addition, different fungi have been also associated with respiratory disease in cattle. *Candida krusei* was determined to be the cause of a bronchopneumonia case in a heifer; fungi isolated from the patient nasal discharge (26) and *Aspergillus fumigatus* isolated from a tracheal tissue and swab and bronchial mucosa caused necrotizing tracheobronchitis in a cow (27). Regardless of the knowledge of fungal species that can cause respiratory disease in humans and cattle, there is a lack of evidence that shows the role of fungal communities in BRD development. Therefore, the objective of this correlative study is to: (i) compare if the nasal mycobiome differs between BRD-affected and visually healthy cattle and (ii) determine bacterial-fungal co-occurrences in both test groups using previously identified bacterial community of these samples (12). Through this work, we expect to identify potential fungal community compositions in healthy animals and fungal species that are associated with BRD incidence which can aid disease identification and treatment.

## Materials and methods

All procedures involving working with animals in this study were approved by the Purdue University Animal Care and Use Committee (protocol #1906001911). A total of 133 Holstein steers approximately 6–7 months old, housed in the same environment were sampled from July to December 2020 at a feedlot (Indiana, United States). Each animal was sampled once throughout the study.

Animals with BRD clinical signs were identified following the DART method that uses depression, appetite loss, respiratory character change and rectal temperature (8) as clinical signs of disease. Animals that exhibited 2 out of 3 clinical signs (not including rectal temperature) were selected and are referred to as BRD animals similar as Centeno-Martinez et al., (12). Once an animal was identified to have BRD clinical signs, one or two apparently healthy pen mates were also selected for nasal swab sampling. Healthy animals were identified as animals that did not present any BRD clinical signs. A total of 75 healthy and 58 BRD animals were included in the study. See Centeno-Martinez et al., (12) for more information about the animal selection criteria.

### Nasal swabs DNA extraction and its gene sequencing

After identifying the healthy and BRD animals, two nasal swabs were collected from each animal. From the nasal swabs, the total DNA was extracted using the DNeasy Blood & Tissue Kit (Qiagen, Germantown, MD, United States) following the protocol described in Holman et al. (2). All the procedures regarding nasal swabs collection and processing before extraction was performed as stated in Centeno-Martinez et al., (12). From the extracted DNA, ITS (Internal Transcribed Spacer) sequencing was performed following the protocol described previously (28). In addition, for each ITS gene sequencing run, 10 known fungal DNA strains (Mycobiome Genomic DNA mix, ATCC®, MSA-1010TM) were used as the positive control (Mock1 and Mock2) and PCR grade water were used as the negative control (Water1 and Water2) for the amplification process. The amplicons were sequenced using Illumina MiSeq Sequencer (2 × 250 paired-end) at the Purdue Genomic Core Facility.

The raw sequence data obtained from the ITS gene amplification were analyzed using Quantitative Insight Into Microbial Ecology (QIIME2) v.2020.2. Using DADA2 (v. 2022.11.1) (29), the forward and reverse sequences were trimmed at position 0, and truncated at position 227 and 156, respectively. This step allowed us to keep sequences with a per-position quality >30. The taxonomy of each sequence was assigned using UNITE v.8.3 databases (30, 31). Then, sequences were rarified to 19,784 reads per sample to calculate the alpha and beta fungal diversity in the nasal swabs. The alpha diversity was estimated in QIIME2 using Observed ASVs and Chao1 to estimate fungal richness, and the Pielou index to estimate evenness and Faith (Faith’s PD) for phylogenetic diversity (32–35). To analyze the fungal beta diversity, Bray–Curtis Dissimilarity Index and Weight UniFrac (36) were calculated and plotted as principal coordinate analysis (PCoA) using R. v 4.0.3.

DESeq (37) was used to determine differentially abundant taxa between BRD and healthy animal, using the unrarefied table (7,050 ASVs) as the input data. Any ASVs with a log2 fold change >2 and statistical significance of *p <* 0.05 were selected as differentially abundant ASV between BRD and healthy animals. In addition, based on the DESEq results, we were able to observe specific *Trichosporon* and *Issatchenkia orientalis* ASVs that were increase on both BRD and healthy group. Therefore, we then determined if the *Issatchenkia* and *Trischosporon* enriched in either in the BRD or healthy group formed subspecies clades. To achieve this, all amplicon sequences assigned to *Issatchenkia* and *Trischosporon*, as well as three reference sequences identified as *Issatchenkia* or *Trichosporon* were obtained from NCBI, were selected to create a phylogenetic tree. Using BLAST, we found that our sequences matched more closely to *Pichia kudriavzevii* sequences. Therefore, for the tree, we selected the ITS region of *Pichia kudriavzevii* ATCC 6258 (38), *Pichia kudriavzevii* isolate 3Y3 (39), and *Pichia kudriavzevii* strain VN9Y (40). For *Trichosporon,* the fungal specie *Trichosporon ovoides* (41, 42), *Trichosporon* sp. isolate mYJh51 (43), and *Trichosporon* sp. Bio4 (44) were selected as reference sequences. *Issatchenkia* and *Trischosporon* sequences were submitted separately to a multiple sequence alignment using MAFFT. To create the alignment, we used an iterative refinement method (global-pair) with weighted sum-of-pairs (WSP) and consistency score with 1,000 iterations (45). Two ASVs assigned to the same genus and within the same family as *Issatchenkia* and *Trichosporon* were used as the outgroups to build the tree. In the case of *Issatchenkia,* the fungal genus *Kazachstania* (ASV1688 and ASV6787, same family as *Issatchenkia*) were selected as the outgroup and for *Trichosporon,* the genera *Vanrija* (ASV6801 and ASV3781, same family as *Trichosporon*) were selected.

### Fungal mock community and PCR negative control sequencing analysis

The raw ITS gene sequencing data from the 10 known fungal strains (Mycobiome Genomic DNA mix, ATCC®, MSA-1010TM) and PCR grade water were used as a PCR negative control were analyzed separately using QIIME v.2020.2. as described above. Using DADA2 (29), the forward and reverse sequences of the fungal mock community were trimmed at position 0, and truncated at position 244 and 184, respectively. The PCR negative control forward sequences were trimmed at position 0 and the reverse at positions 244 and 170. To evaluate the fungal mock community sequencing quality, we used the function evaluate_seqs in QIIME2 (v.2020.2) function (46). Two fungal mock community technical replicates were amplified and sequenced to target the ITS gene.

### Statistical analysis for ITS gene sequencing data

Fungal alpha diversity metrics (Observed ASVs, Chao1, Faith’s PD, and Pielou) were analyzed with a Generalized Linear Mixed Model with a random factor structure specified to only include random slopes using the afex package (47). In the model, clinical status (BRD or healthy) was included as the fixed factor, and pen number as the random factor. The animal age and date of samples were included as continuous factors in the model. We then checked for the normality of the residuals and homogeneity of variance. Normality was checked using a Shapiro–Wilk normality test with a significance of *p <* 0.05. We square-transformed dependent variables when assumptions were not met. Only the dependent variables Observed ASV and Chao1 were square-transformed. The *F* values were reported with their corresponding degrees of freedom associated with the model. In the case of Faith’s PD and Pielou, these dependent variables did not meet normality assumptions after transformation (log transformation, square transformation, and cubic transformation). Thus, a non-parametric test, Mann–Whitney’s test, using the ‘wilcox.test’ function in R (48, 49) was performed to only include the effect of clinical status (BRD vs. healthy) in mycobiome phylogenetic diversity (Faith’s PD) and evenness (Pielou).

To determine the difference in the fungal community structure (beta diversity) between BRD and healthy animals as well as based on the date of sampling, a permutational multivariate analysis and variance test (PERMANOVA) of the Bray–Curtis dissimilarity and Weighted UniFrac distances was performed using the ‘adonis’ function from the vegan package (50). To determine the date of sampling effect, nasal swabs were grouped by 2 months from July to December due to the small sample size for some months: July–August (BRD, *n* = 24 and healthy, *n* = 30), September–October (BRD, *n* = 17 and healthy, *n* = 21), and November–December (BRD, *n* = 15 and healthy, *n* = 24) and season. Group was included as an independent factor in the model. In addition, a dispersion test, using as input the distance matrices determined by Bray–Curtis dissimilarity and Weighted UniFrac, was performed to analyze the average distance of the samples from the two groups (healthy or BRD animals) using the function ‘betadisper’ from the vegan package (50) followed by a permutation test of multivariate homogeneity of groups dispersion. In this study, statistical significance was defined as *p <* 0.05.

### Classification of healthy and BRD animals based on bacterial and fungal community

A random forest analysis (RF) using the package randomForest in R v.40.0.3 was performed using the unrarefied fungal ASV table (7,050 ASVs). As a first step, the fungal ASV table was filtered to only include ASVs with an abundance >0.0001 and rarified to 40,420 reads per sample. After applying the filtering step, a total of 1,109 ASVs were used to build the RF model. Following this step, the samples were divided into training (60% of the total samples, *n* = 79, containing 33 BRD samples and 46 Healthy samples) and testing set (40% of the samples, *n* = 54, containing 25 BRD samples and 29 healthy samples). The RF model was built from the training set using 17 trees; with this number, it was possible to achieve the lowest error rate (Supplementary Figure S2). In addition, the training set was used to identify fungal ASV with the highest importance for the model accuracy based on a 10-fold-cross-validation (‘rfcv’ function). This step indicates how much accuracy the model loses if the ASV is not included (51) (Supplementary Figure S2A). The model was then applied to the testing set. Additionally, a RF analysis using the quantification of BRD-pathobiont abundance, and data previously published (12) was used to compare how the bacterial and/or fungal community can classify BRD and healthy animals. The RF model including the BRD-pathobionts abundance data was built from the same training set used in the fungal RF model using 35 trees, and then, it was applied to the same testing set. To determine the classification accuracy of the two models (fungal community and BRD-pathobiont abundance), the sensitivity (Eq. 1), specificity (Eq. 2), and misclassification rate of the models were calculated. To calculate the misclassification rate, the animal visual diagnosis and the RF classification for the sample were compared. Lastly, the samples that agreed with the classification between the visual diagnosis and the RF classification were identified for each of the RF models: fungal community and BRD-pathobiont abundance. For each of the two RF models, the factors that had the highest impact in classifying the samples were selected based on the mean decrease in accuracy (Supplementary Figure S2B).

$$\mathrm{Sensitivity}=\frac{\#\mathrm{of true positives}}{\#\mathrm{of true positives}+\#\mathrm{of false negatives}\;}\;\left(\mathrm{Equation}\;1\right)$$

$$\mathrm{Specificity}=\frac{\#\mathrm{of true negatives}}{\#\mathrm{of true negatives}+\#\mathrm{of false positives}}\;\left(\mathrm{Equation}\;2\right)$$

### Fungal and bacteria co-occurrence analysis

A co-occurrence analysis was performed to identify fungal and bacterial ASVs that co-occur in the same sample. In this analysis, it was possible to identify fungal and bacteria ASVs that coexist in the same sample (positive associations) or do not co-exist (negative associations). Both ASV tables, fungal (7,050 ASV) and bacterial [(12), 18,010 ASVs] were pruned to only utilize ASVs with an abundance >0.0001 and rarified to 40,420 reads per sample. A total of 1,236 bacterial ASVs and 1,109 fungal ASVs were utilized as the input. Additionally, both ASVs tables were transformed to a binary format: presence (1) and absence (0) and then combined into one single table; thus, each sample contained bacterial and fungal ASVs. The co-occurrence analysis was performed separately for the BRD and healthy animals using the package cooccur (52) in R v.4.0.3. The positive associations were identified as ASVs pairs that were significant (*p <* 0.05), present in more than 60% of the samples (BRD > 37 samples and healthy >44 samples) and with a probability >0.75 that the two ASVs (bacterial and fungal) will co-occur in the same sample. In the case of the negative associations, it was required that the ASV pairs be significant (*p <* 0.05) with a probability of <0.05 that the two ASVs (bacterial and fungal) would co-occur in the same sample.

In addition, any positive fungal ASV pair combination with BRD-pathobionts (*H. somni, P. multocida, M. haemolytica,* and *M. bovis*) were selected with a significance of *p <* 0.05. This allowed the identification of fungi associated with BRD-pathobionts in the nasal cavity of healthy and BRD animals.

## Results

### Fungal taxonomy composition in the mock community and PCR negative control sequencing analysis

A total of 178,415 sequences were identified and were classified into 28 amplicon sequence variants (ASVs) in the fungal community used in the study. After comparing the fungal mock community with the 10 known fungal strains DNA, 9 out of 10 fungal strains were identified. In the case of the PCR negative control (water), a total of 105,882 sequences were identified and classified into 21 ASVs. Interestingly, both water samples presented a different fungal composition. Water1 was mostly composed of *Cyberlindnera jadinni* and *Oidiodendron truncatum* (>50% of community composition) whereas Water2, was composed of *Fusarium sporotrichioides* (>50% of the community composition, Supplementary Figure S1).

### Fungal nasal taxonomy composition in healthy and BRD animals

**Figure 1:**
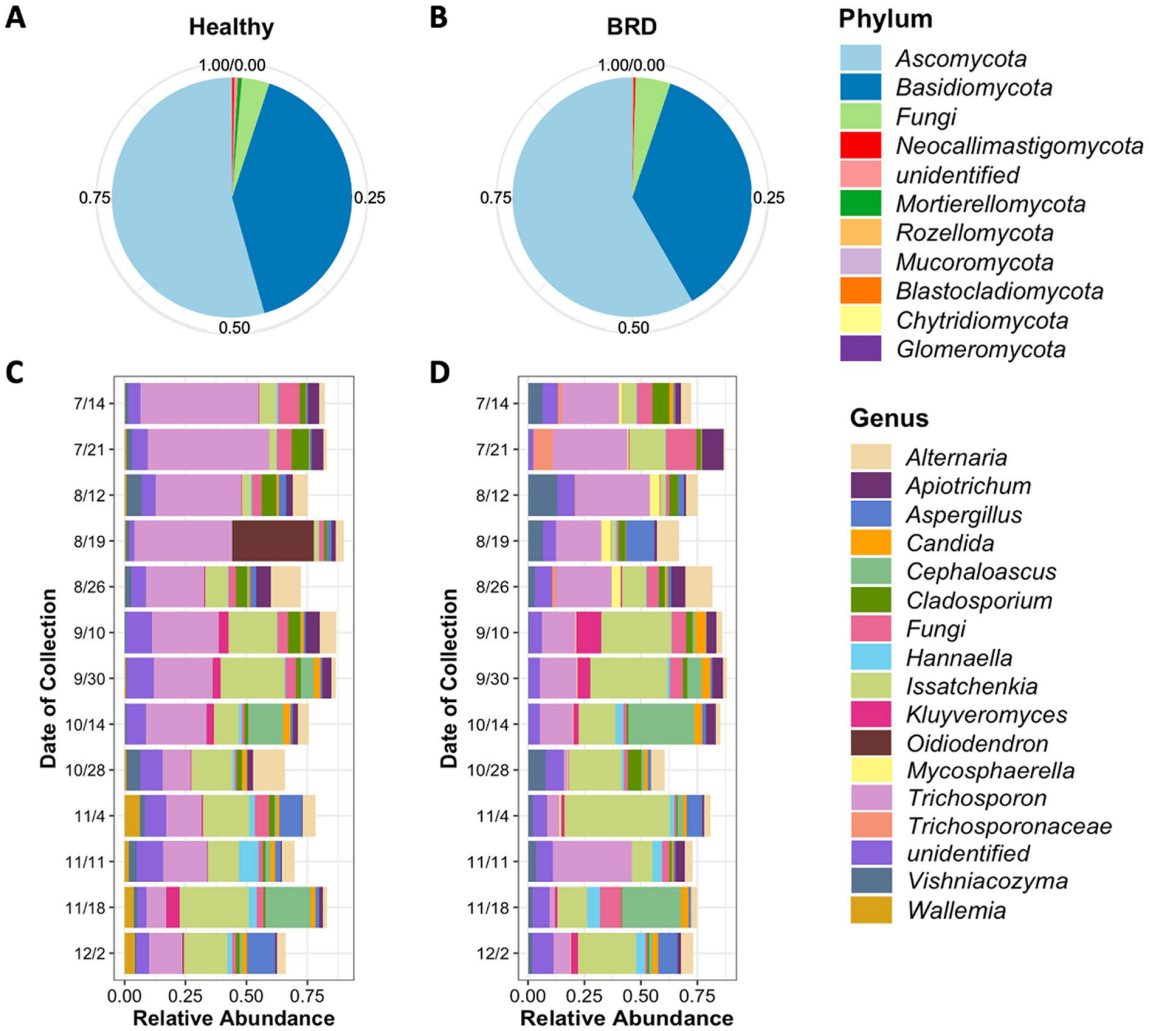
Average relative abundance of fungi present in the nasal cavity of healthy and BRD-animals. Pie charts represent the relative abundance at phylum level of fungi present in the healthy (*n* = 73) **(A)** and BRD animals (*n* = 56) **(B).** Bar plots represent the 15 most abundant genera in the healthy **(C)** and BRD animals **(D)** sampled from July (7/14) to December (12/2) 2020 (date of collection).

A total of 8,481,537 fungal sequences were obtained before the denoising step (DADA2) and 6,092,893 after denoising from healthy and BRD cattle nasal swabs. A total of 7,050 fungal ASVs from 11 different phyla were observed in the nasal cavities of healthy animals (*n* = 73). From these results, the fungal phyla with the higher relative abundance were *Ascomycota* (~54.36%), and *Basidiomycota* (38.81%), followed by unclassified fungi (3.71%). No other phyla had a relative abundance >1% (Figure 1A; Supplementary Table S1). At the genus level, a total of 492 different genera were observed in the nasal cavity of healthy animals. However, two genera had the highest relative abundance in the nasal cavity: *Trichosporon* (25.93%) and *Issatchenkia* (14.15%), followed by unidentified fungi (7.14%) and *Alternaria* (5.26%). Any other fungal genus identified had a relative abundance of <5% (Figure 1C; Supplementary Table S2).

Fungal community composition was also characterized in BRD animals (*n* = 56). A total of 11 phyla fungal were identified in the study, from which the phyla *Ascomycota* (58.34%), and *Basidiomycota* (36.49%) followed by unidentified fungi (4.72%) were the most abundant in BRD animal swabs. Any other phylum group observed in the study had a relative abundance <1% (Figure 1B; Supplementary Table S1). A total of 492 different genera were observed in the nasal cavity of BRD animals. From these results, again the genera *Issatchenkia* (19.59%), and *Trichosporon* (18.88%) were the most abundant followed by unidentified fungi (11.0%) and *Alternaria* (4.50%). All other genera had an average relative abundance of <4% (Figure 1D; Supplementary Table S2).

**Figure 2:**
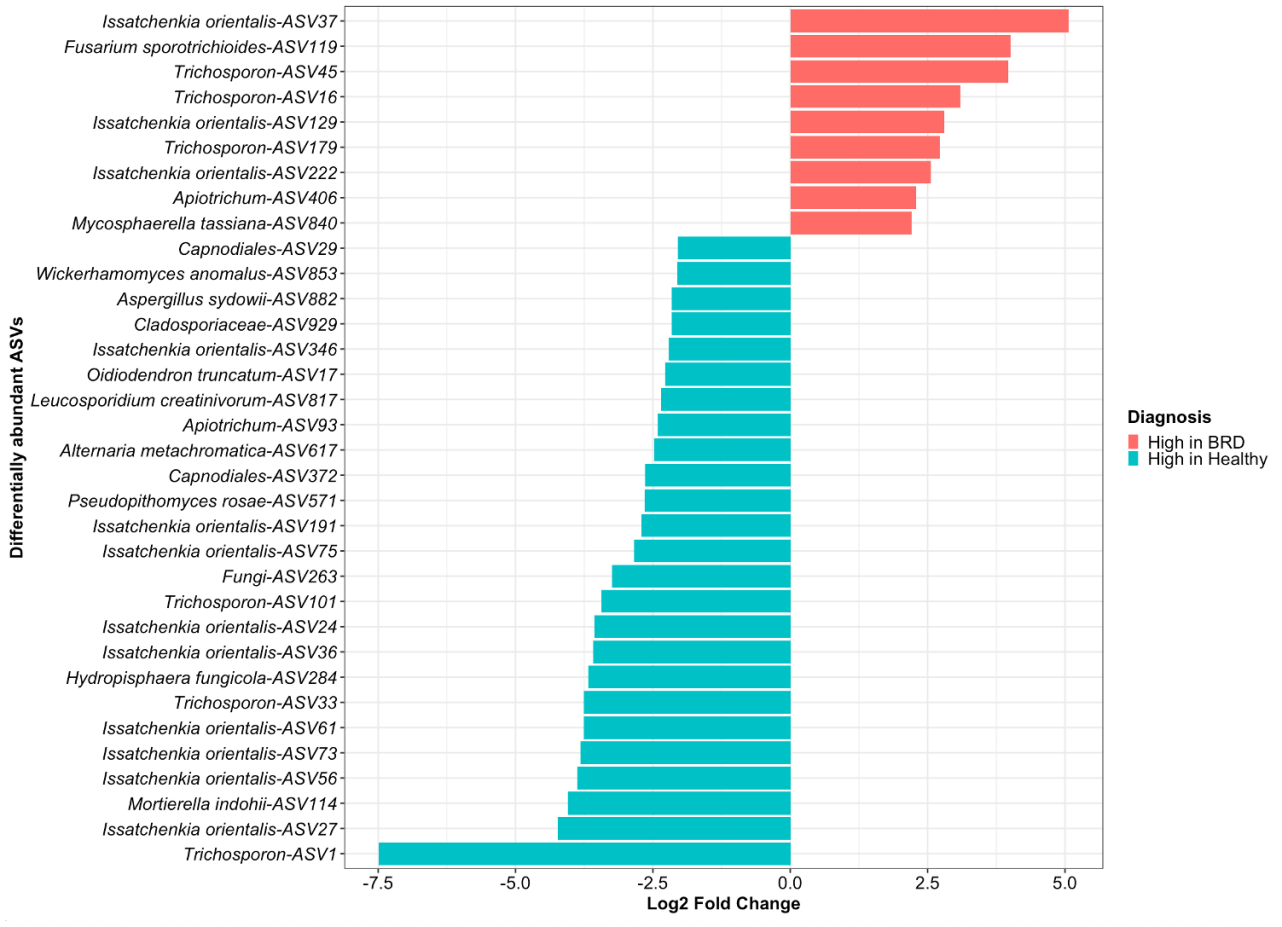
Differentially abundant fungal taxa (ASVs) between animals with BRD clinical signs (BRD) and healthy animals. Bar plots indicate fungal ASVs with a log2 fold change >2 were enriched in BRD animals and a log2 fold change < − 2 indicate those decreased in the BRD animals.

A total of 34 differentially abundance fungal ASVs were observed, from which 9 were significantly increased in the BRD group and 25 in the healthy group (log2 fold change >2, *p <* 0.05, Figure 2). From the differential abundant ASVs, *Issatchenkia orientalis*-ASV37 and *Fusarium sporotrichioides*-ASV119 presented the highest log2 fold change in the BRD group (5.06 and 4.00, respectively). Meanwhile, in the healthy group, *Trichosporon*-ASV1 and *Issatchenkia orientalis*-ASV27 had the highest log2 fold change (7.48 and 4.22, respectively). A total of 12 ASVs classified as *Issatchenkia orientalis,* 6 classified as *Trichosporon*, and 2 classified as *Apiotrichum,* were significantly increased in one of the groups. No other genera had ASVs that were significantly increased or decreased in both groups. Differentially abundant fungi in the healthy group were assigned to more genera than those in the BRD group. Of the 25 differentially abundant fungal ASVs in the healthy group, 12 were not classified as *Issatchenkia* or *Trichosporon*: *Mortierella indohii, Hydropisphaera fungicola,* unclassified fungi, *Pseudopithomyces rosae,* two ASVs classified as *Capnodiales, Alternaria metachromatica, Leucosporidium creatinivorum, Oidiodendron truncatum,* unclassified *Cladosporiaceae*, *Aspergillus sydowii,* and *Wicherhamomyces anomalus.* On the other hand, only 2 out of 9 differentially abundant fungi in the BRD group were not classified as *Issatchenkia* or *Trichosporon*: *Mycosphaerella tassiana* and *Fusarium sporotrichioides* (Figure 2).

We then determined if the *Issatchenkia* and *Trischosporon* enriched in either in the BRD or healthy group formed subspecies clades. We selected all amplicon sequences assigned to *Issatchenkia* and *Trischosporon*, as well as three reference sequences identified as *Issatchenkia* or *Trichosporon* were obtained from NCBI to create a phylogenetic tree. The ITS gene-based phylogenetic analysis across *Issatchkenia* sp. ASVs identified that six ASVs (numbers 75, 191, 27, 61, 36, and 73), which all had a high abundance in the healthy group (log2 fold change >2, *p <* 0.05, Figure 2) clustered within the same clade (Supplementary Figure S3). On the other hand, three of the ASVs with high abundance in the BRD animals (numbers 129, 222, and 37) clustered on the same branch as two ASVs with high abundance in the healthy animals (numbers 24 and 56, Figure 2). Interestingly, the *Pichia kudriavzevii* sequences retrieved from the BLAST search and used as the reference sequences for the tree, clustered separately from our obtained *Issatchenkia orientalis* ASVs.

The *Trichosporon* sp. tree using the ITS gene-based phylogeny revealed two lineages on the same branch that included *Trichosporon* ASVs, number 16 and 179, with high relative abundance in the BRD animals (log2 fold change >2, *p <* 0.05, Supplementary Figure S4). ASVs 101 and 1 with high abundance in the healthy group (log2 fold change >2, *p <* 0.05, Figure 2) clustered separately and distinctly from ASVs enriched in healthy animals. Two *Trichosporon* ASVs that were increased in the BRD, and healthy group, ASV33, and ASV45, respectively, clustered on distinct small branch in the tree. On the other hand, the *Trichosporon* reference sequences clustered with other *Trichosporon* ASVs observed in our study but did not cluster with any differentially abundant *Trichosporon* ASVs (Supplementary Figure S4).

### Nasal fungal alpha and beta diversity

In this study, the fungal richness estimated by Observed ASVs and Chao1, phylogenetic diversity by Faith’s PD, and fungal community evenness by Pielou were not different between BRD and healthy animals nor by sample collection date (*p* > 0.05).

**Figure 3:**
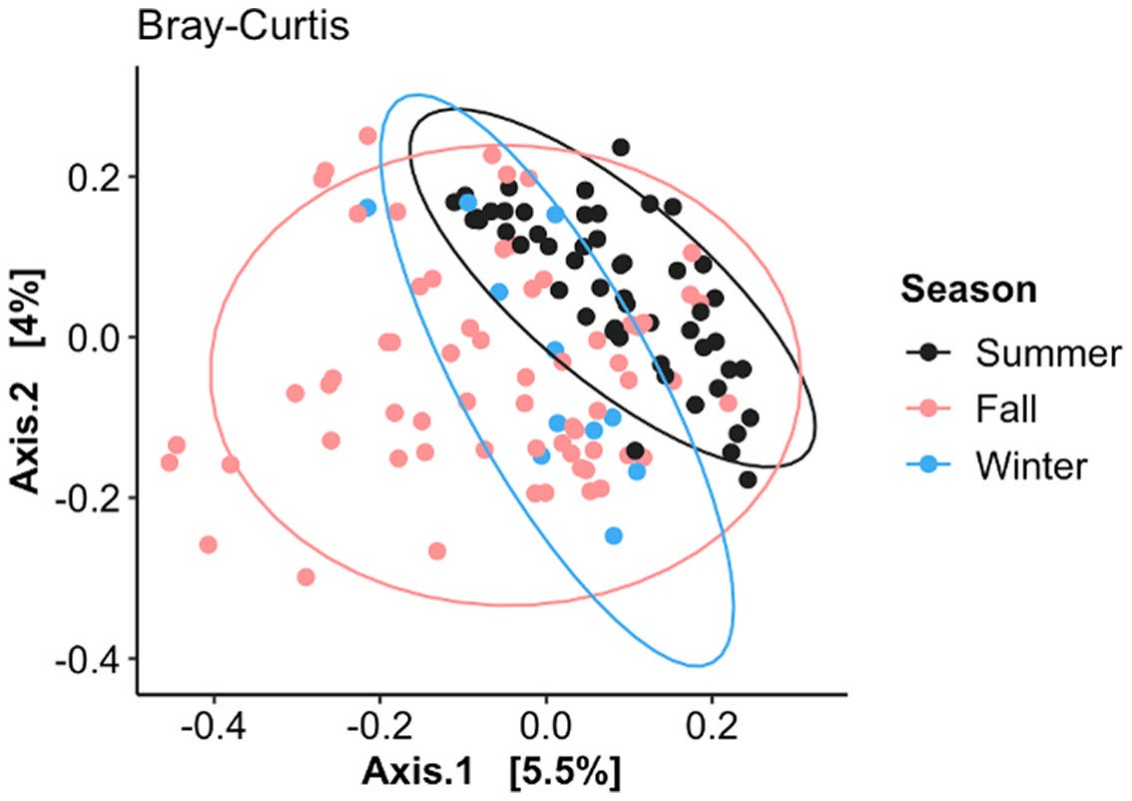
Principal coordinate analysis (PCoA) of Bray–Curtis dissimilarity based on when the nasal samples were collected (July–August, September–October, and November–December). Ellipses indicate a 95% confidence interval of individuals belonging to each season. Axis 1 represents the axis that explains the greatest amount of the variation followed by Axis 2.

The nasal fungal community structure or distance between the BRD and healthy animals (beta diversity) as determined by Bray–Curtis dissimilarity (F2, 125 = 1.23, *R*^2^ = 0.019, *p* < 0.006; Figure 3) was different based on when the nasal swab was collected (seasonal effect), but no difference was observed between BRD animals and the healthy group (*p* > 0.05). Nasal swabs were grouped by date of sampling from July to December 2020 (Figure 3): July–August (BRD, *n* = 24 and healthy, *n* = 30), September–October (BRD, *n* = 17 and healthy, *n* = 21), and November–December (BRD, *n* = 15 and healthy, *n* = 24). This grouping allowed us to identify if season affects the nasal fungal community structure. Interestingly, the pairwise testing between the three seasonal groups demonstrated that the fungal community was different between the July–August and November–December groups (Summer vs. Winter season, F1, 87 = 1.49, *R*^2^ = 0.02, *p* = 0.002). Additionally, no difference was observed in the fungal nasal community as predicted by Weighted UniFrac based on the season or between the two groups. In addition, no difference in the dispersion of the samples as predicted by Bray–Curtis dissimilarity and Weighted UniFrac was observed between the season group centroids and between BRD animals and the healthy group (*p* > 0.05).

### Classification of BRD-affected and healthy animals using the fungal nasal community and BRD-pathobionts abundance

**Figure 4:**
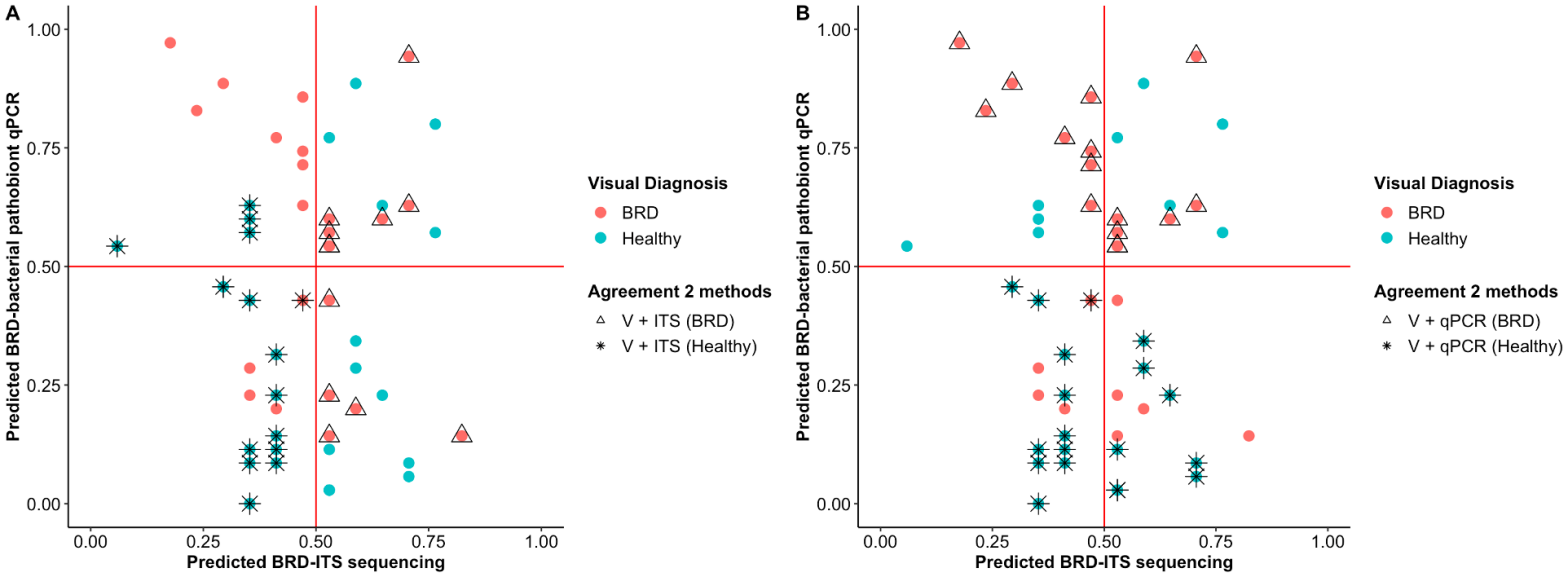
Probability of classifying animals as BRD or healthy (<0.5 = healthy, >0.5 = BRD) using Random Forest analysis. Classification of the animals based on the nasal fungal community composition (ITS table) **(A)** and quantification of BRD pathobionts (qPCR) **(B)**. The color indicates the initial animal classification based on the visual diagnosis of BRD. Shape indicates if the animal classification agreed between the two methods: visual classification based on BRD clinical signs (V) and the fungal community composition (ITS Table) or visual classification based on BRD clinical signs (V) and BRD-pathobiont abundance (qPCR). In one case, two animals (one healthy and one BRD) are overlapping at ITS and qPCR predicted scores of 0.45 and 0.4, respectively.

Random forest (RF) analysis was used to predict animal health status based on (1) nasal fungal community and (2) abundance of BRD-pathobionts. To create the model, 60% of the samples (*n* = 79) were utilized to train the random forest model and 40% of the samples (*n* = 54) were used for the testing set. In the testing set, 29 animals were identified as healthy based on the BRD clinical signs and 25 as BRD. Once the RF analysis using the fungal community data was applied to the testing set, 12 animals were correctly predicted as BRD and 15 as healthy animals with an accuracy of 51.9%, a sensitivity of 48%, and a specificity of 55% (Figure 4A). When the abundance of BRD-pathobionts in the nasal cavity was utilized to classify the animals, 15 animals were correctly predicted as BRD and 19 as healthy with an accuracy of 65.3%, sensitivity of 62.5%, and specificity of 67.8%. In addition, 7 out of 25 visually identified as BRD and 11 out of 29 visually identified as healthy agreed with the fungal community and BRD-pathobionts abundance classification. Additionally, 4 animals visually identified as healthy were classified as BRD by the fungal community and BRD-pathobionts RF models, and 4 visually BRD animals were classified as healthy by the two models. Another piece of information that both RF models provided was the capacity of each model to predict the health status of one animal when the other method fails to make an accurate prediction. In this study, the fungal community agreed with the visual identification for 5 BRD and 4 healthy animals that the BRD-pathobiont abundance did not identify. A similar result was observed for the BRD-pathobionts RF model, in which 8 BRD and 7 healthy animals agreed with the visual identification but were not predicted by the fungal community (Figure 4B).

### Nasal bacterial and fungal co-occurrence

**Figure 5:**
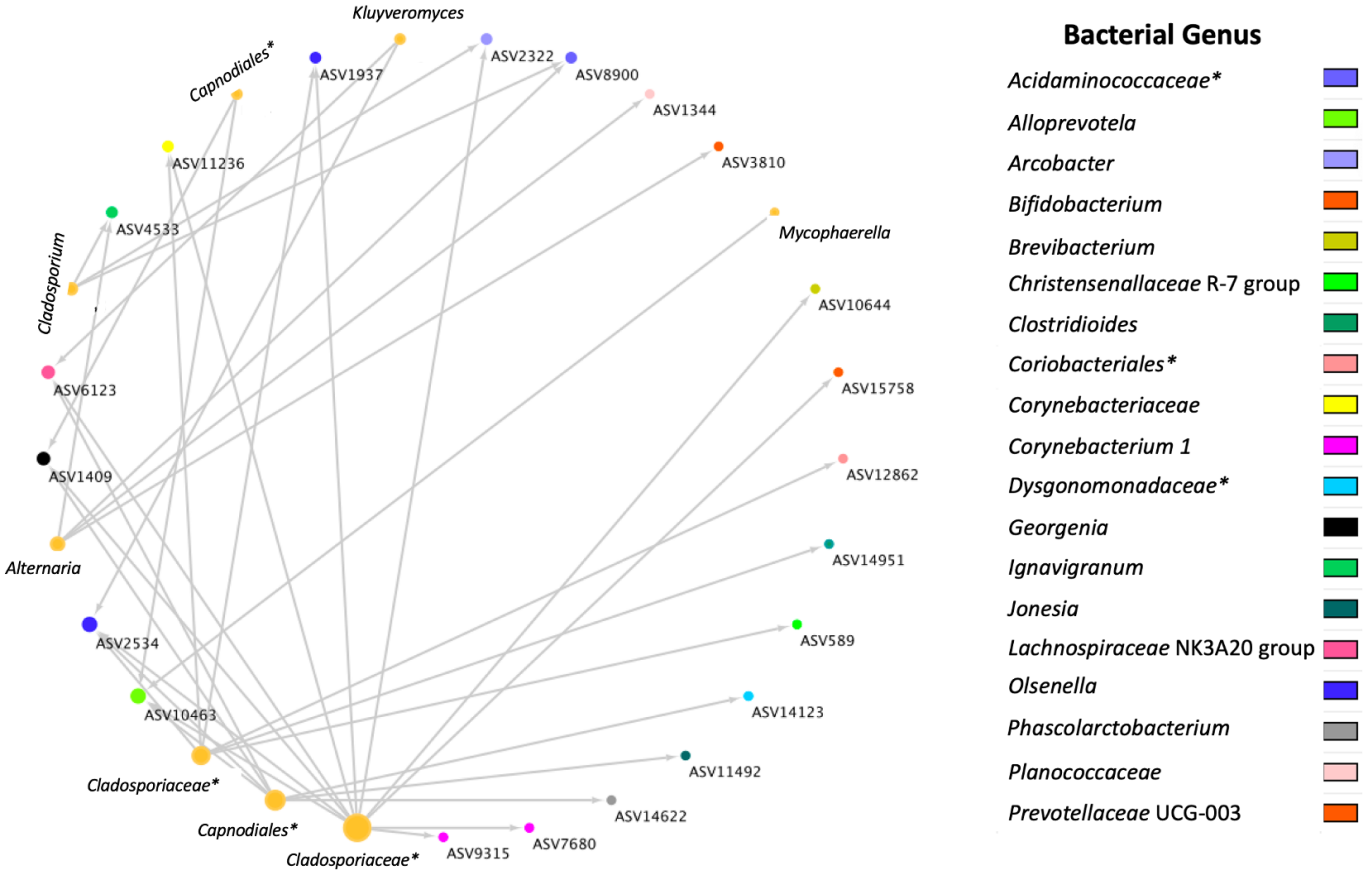
Positive bacterial and fungal ASV co-occurrence in the BRD group (*n* = 36) with a probability of occurrence in the same sample >0.75. Circle sizes represent the total associations for each ASV (min 1 max 11 associations). Color indicates the bacteria genus classification. Lines indicate co-occurrence between ASVs. *Indicates taxonomy not identified at genus level.

**Figure 6:**
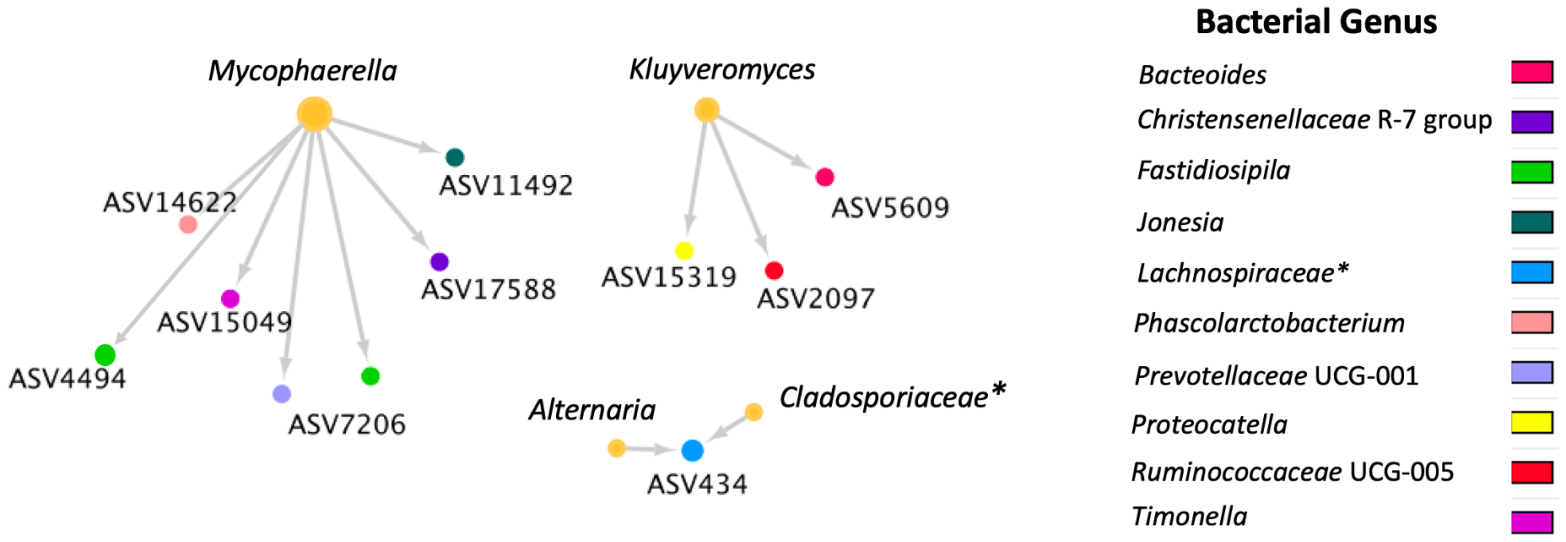
Positive bacterial and fungal ASV co-occurrence in the healthy group (*n* = 11) with a probability of occurrence in the same sample >0.75. Circle sizes represent the total associations for each ASV (min 1 and max 6 associations). Color indicates the bacteria genus classification. Lines indicate co-occurrence between ASVs. * Indicates taxonomy not identified at genus level.

A total of 36 positive co-occurrence associations were observed in the BRD group (Figure 5), and 11 in the healthy group (Figure 6). Interestingly, 4 fungal ASVs classified as *Mycophaerella* (ASV14)*, Kluyveromyces* (ASV6)*, Alternaria* (ASV2), and at the family level *Cladosporiaceae* (ASV4) were positively associated with bacteria in both groups. On the other hand, a total of 733 negative associations were observed in the BRD group and 1,543 in the healthy group. In the BRD group, one fungal ASV presented the most negative associations (>30 negative co-occurrences) with bacteria: *Rhodotorula kratochvilovae.* In the case of the healthy group, 7 fungal species presented the most negative associations with bacteria ASVs (>30 co-occurrence): *Ophiosphaerella aquatica, Curvibasidium cyaneicollum, Aspergillus caesiellus, Penicillium melanoconidium, Wickerhamomyces anomalus, Leucosporidium creatinivorum, Cyverlindnera culbertsonii,* and *Cyberlindnera rhodanensis*. Additionally, we sought to identify fungal ASVs that were associated with BRD-pathogens: *Mycoplasma bovis, Mannheimia haemolytica, Pasteurella multocida,* and *Histophilus somni* in the nasal cavity of BRD and healthy animals. However, no significant positive co-occurrences between fungi and BRD-pathobionts were identified (*p >* 0.05).

## Discussion

While it has been well established that bacterial pathobionts such as *Pasteurella multocida, Histophilus somni, Mannheimia haemolytica,* and *Mycoplasma bovis* are strongly related with BRD (2, 12–15), the role of fungi in BRD development is understudied. It is important to recognize that fungal community or mycobiome may interact with other microbes impacting the host’s health and immune response of the host (53). Thus, in this study, nasal swabs collected from BRD and healthy pen-mates animals (that also were used in a companion study (12)) to identify differences in the nasal mycobiome due to healthy status and to determine cross-kingdom interactions between bacteria and fungi present in the nasal environment. Based on our results, *Trichosporon* sp. and *Issatchenkia* sp. were the most abundant genera regardless of disease diagnosis, and no major difference in the fungal alpha and beta diversity were found due to health status. Additionally, the classification of BRD or healthy pen-mates using ITS sequencing agreed more than 50% of the time with the visual diagnosis. This classification also identified different BRD animals that the qPCR method missed and more positive and less negative co-occurrence between fungi and bacteria in BRD than healthy animals. This study will benefit the field of BRD research as it represents the first time that the nasal fungal community was analyzed in healthy and BRD animals and can help set an initial baseline in the potential role of these fungal communities with BRD development. While fungal community composition does not seem to be important factor for BRD occurrences in this farm, more research is needed across different locations to determine the importance of fungi on BRD development and to increase the knowledge of how fungi interact with bacteria in the cattle’s nasal cavity.

### Description of the healthy and BRD fungal community

Previous characterization of the respiratory tract microbiome of cattle has focused on the bacterial community composition present at different sites of the respiratory tract (54–56). The term “microbiota” can be defined as all microorganisms of the environment including commensal and pathogenic members (57). While bacteria are critical members of the microbiota, cross-kingdom interactions (bacteria, fungi, viruses, and archaea) can prove critical to understand the dynamics in the microbiome (53). Fungal community analysis has traditionally relied on culture-based methods. Nonetheless, the advancement in deep-sequencing technologies has provided the opportunity to unveil fungal community composition across multiple environments such as human and animal barrier surfaces (58). Most approaches rely on the sequencing of the internal transcribed spacer (ITS) region and using computational analysis to characterize the fungal community in the environments (28, 58). With the combination of new sequencing methods and culture-based approaches, the fungal “mycobiome” composition of several mammalian (mostly human) mucosal environments, including the respiratory tract, vaginal tract, urinary tract, oral cavity, intestine, skin, breast, and breastmilk have been characterized (53, 59–63). The most common fungal genera observed in the nasal cavity were *Aspergillus, Cladosporium, Penicillium, Candida parapsilosis*, *Saccharomyces cerevisiae, Malassezia,* and *Pleosporales.* A culture-dependant study of the horse nasal mycobiome identified the presence of *Candida* sp. as a normal commensal fungi in healthy animals (64). Interestingly, at the phyla level, it was observed that the majority of fungal sequences were classified as *Ascomycota* followed by *Basidiomycota* fungi (62). Regardless of the advancements in understanding the commensal mycobiome composition in humans, in the dairy and beef industry, most fungal studies had a focus on pathogenic fungi and their relationship with disease development leaving aside the characterization of commensal mycobiome. As an example, *Candida* spp., have been related to several diseases in cattle such as mastitis, abortion, otitis externa, gastrointestinal infection, and disseminated candidiasis (23). One species, *Candida krusei,* has been linked with bronchopneumonia (26). Therefore, one objective of our study was to characterize the nasal fungal microbiota of healthy cattle as a means to understand the nasal fungal community and potentially use these results to understand the dynamic between fungi, bacteria, and potentially viruses in BRD research. In our study, the nasal cavity of healthy animals was mostly composed of the fungal phyla *Ascomycota* and *Basidiomycota*, with similar results as observed in Botero et al., (62). At the genus level, *Trichosporon* sp. and *Issatchenkia* sp. were the most abundant. We could also identify the presence of “known” human commensal mycobiota (e.g., *Aspergillus, Candida, Penicillium, Cladosporium, Malassezia*), but their relative abundance in the nasal cavity of healthy animals was lower than 1% of the community. Interestingly, the genus *Trichosporon* has been linked to mucosal or cutaneous infections in the immunocompromised host (65, 66). On the other hand, the genus *Issatcheknia* was also prevalent and abundant in our bovine nasal samples. The species *I. orientalis*, also identified as *Candida krusei*, *Saccharomyces krusei* (67), and *Pichia kudriavzeveii* was previously isolated from tracheal and bronchial samples of a one-year-old heifer with bronchopneumonia (26). *Pichia kudriavzeveii* was recently identified by the World Health Organization as human fungal pathogen with medium priority group because drug resistance can cause treatments challenges for invasive acute and subacute systemic infections (68). Even though the presence of these two fungal genera have been related to disease in humans and cattle, more research is needed to understand if *Trichosporon* and *Issatchenkia* are commensal organisms in the nasal cavity of healthy animals and if they can become pathogenic when the host immune system is compromised.

In our study, we observed that the nasal fungal community in BRD animals had a similar composition at the phylum level as the healthy animals, with *Ascomycota* and *Basidiomycota* dominating the community. At the genus level, *Issatchnekia* sp. was the most abundant followed by *Trichosporon* sp. One limitation of our results was the high relative abundance of ASVs that did not match with the fungal UNITE database (30) (noted as unidentified ASVs), highlighting the breadth of fungal members that have not yet been characterized. In addition, we observed that *Alternaria*, *Vishniacozyma, Apiotrichum,* and *Cephaloascus* were common genera in the nasal cavity. We also identified known human “commensal” fungi (e.g., *Aspergillus, Candida, Penicillium, Cladosporium, Malassezia*) in the BRD animals but reflected a relative abundance of 1%. After comparing the fungal genus profile between the BRD and healthy animals, it was surprising to detect that the *Issatcheknia* genus was more abundant in the BRD animals and the *Trichosporon* genus were more numerically abundant in the healthy animals. From these results, we expect to set a starting point in understanding and identifying the “commensal” respiratory mycobiome composition specifically in cattle, and how it can be disturbed when disease occurs. In addition, it was observed that the succession in the healthy mycobiome seems more predictable than in BRD animals.

### Difference at *Issatchenkia orientalis* and *Trichosporon* sub-species level can be a potential indicator of disease status

One of our goals is to detect changes in the microbiome that could explain a difference between two groups being compared (e.g., health status). In previous studies, it was identified that BRD animals had a significant increase in the relative abundance of the bacterial member *Mycoplasma* spp., *Trueperella pyogenes,* and the genera *Biberstinia, Streptococcus*, and *Moraxella,* members that have been related with BRD development (12, 69, 70). On the other hand, healthy animals had a higher relative abundance of ASVs classified as *Clostridium sensu stricto* 1, one unclassified *Moraxellaceae, Mycoplasma bovirhinis* and *Moraxella boevrei* DSM 14,165, previously classified as commensal bacteria in the nasal cavity (12). These results can be further utilized in the process of disease diagnosis. In our study, a total of 34 fungal ASVs were differentially abundant between healthy and BRD animals. From these fungal species, 12 were classified as *Issatchenkia orientalis* and 6 as *Trichosporon* sp. As previously mentioned, members of these two fungi genera have been related to respiratory disease in humans and cattle (26, 65, 66). Nonetheless, different ASVs from these genera were significantly increased in both groups. In recent years, BRD research has highlighted that within BRD-pathobiont species (*P. multocida, H. somni, M. haemolytica,* and *M. bovis*), there are serotypes or subgroups with more pathogenic capacity than other serotypes. Studies revealed that *M. haemolytica* serotypes A1 and A6 are related to bovine pneumonic pasteurellosis (71, 72) and *P. multocida* serotype A3 is a common strain isolated from BRD cases (73, 74). Therefore, we determined if the *Issatchenkia orientalis* and *Trischosporon* sp., ASVs enriched in either the BRD or healthy groups had a genetic or phylogenetic relationship within them that could be interpreted as potentially related with BRD. In our study, there was a clear clade separation of the *Issatchenkia orientalis* and *Trischosporon* sp. potentially indicating a link with health status based on phylogenetic relationship. These phylogenetic trees were created utilizing ITS gene sequencing data. A more detailed study including *Issatchenkia orientalis* and *Trischosporon* sp., the complete genomes would allow greater insight to determine if subspecies clades in these two genera contain the more pathogenic strains.

### Specific changes in fungal members between BRD and healthy animals could help in disease diagnosis

In this study, a difference in relative abundance was observed in specific members of the BRD and healthy nasal mycobiome community. *Fusarium sporotrichioides* was increased in the BRD group, while *Mortierella indohii* and *Hydropisphaera fungicola* were decreased in the BRD group. *Fusarium* species including *F. sporotrichioides,* which was increased in the BRD group, are considered toxic fungi due to the capacity of producing mycotoxins in animal feedstuffs (75, 76), but have not been linked with respiratory diseases. On the other hand, *Mortierella* species can be isolated from diverse environments like soils, caves, and rhizospheres (77, 78). However, there are specific species such as *M. wolffii* that are known to cause bovine mycotic abortion, hepatitis, meningoencephalitis, pneumonia, and systemic mycosis in cattle (78). Interestingly, *M. indohii,* a species that was more abundant in the healthy group, has only previously been isolated from a plant root, and animal feces (79). Lastly, the fungi *Hydropisphaera fungicola,* also with high abundance in healthy animals, had been only isolated on decaying leaves of *Populus trichocarpa* acting as a parasite in Idaho, United States (80). No association of these fungi has been described with respiratory disease or identified as a commensal member of the cattle nasal cavity.

In addition to disease detection based on visual clinical signs, utilization of the bacterial community may become an important tool in the process of identifying BRD. It has been observed that the abundance of BRD-pathobionts in the nasal cavity, can be utilized to classify the animal’s health status using machine learning algorithms (12). However, to our knowledge, no study has utilized the mycobiome community as means to classify and detect BRD cases. In our study, a comparison of the nasal mycobiome community agreed at about 52% with the classification of healthy and BRD animals based on the visualization of clinical signs, when the abundance of BRD-bacterial was included, 65.3%, respectively. Even though the fungal classification rate was lower than the BRD-pathobiont classification rate, it allowed the identification of visually-diagnosed BRD and healthy animals that failed to be classified based on the bacterial pathobiont method. It was observed that the relative abundance of specific fungal taxa in the nasal cavity can be used to classify disease status. Nonetheless, these results were observed by utilizing the ITS sequencing data and a different method should be used to quantify the abundance of these fungal species more accurately in the nasal cavity of BRD-affected and healthy animals.

### Nasal mycobiome community affected by season

In this study, the fungal community structure was affected by season, specifically when comparing samples collected in Summer (July–August) compared to those collected in Winter (November–December), regardless of the disease status. It has been observed that meteorological parameters, such as differences in season mediated by temperature change, can influence the abundance of fungal aerosols (81–83). Interestingly, in a study utilizing sinonasal samples from healthy human subjects, the fungal community was altered between seasons (59). Therefore, it is possible that in our study, changes in environmental conditions could have contributed to a different fungal community structure from the summer to winter of 2020. Unfortunately, no other study has investigated the effect of environmental temperature on the nasal fungal community in cattle.

### BRD animals had more fungal and bacterial co-occurrence than healthy animals

Microbes such as bacteria and fungi are abundant in nature and host environments, leading to multiple cross-kingdom interactions that can provide benefits or disadvantages to the community and the host. The interactions can determine which species will grow and survive (84). Cross-kingdom interactions between fungi and bacteria can be classified into physical interactions, alteration of host immune response, and interactions *via* secreted molecules (84). Therefore, the analysis of inter- and cross-kingdom interactions represents a crucial piece of information needed for the understanding of changes in the environment and even, disease development. In a companion study, an inter-kingdom interaction between bacteria present in the nasal cavity of BRD and healthy animals highlighted that BRD animals had a decrease in bacterial co-occurrence compared to healthy animals (12). We previously concluded that the decrease in bacterial co-occurrence was linked to a decrease in bacterial diversity compared to the healthy animals which can be an indicator of microbial dysbiosis in the nasal cavity of BRD animals (85). Nonetheless, microbial dysbiosis could not be determined as the cause of disease development (86).

To our knowledge, no other study had investigated cross-kingdom interactions between fungi and bacteria in animals with BRD. However, the interactions between specific fungal (such as *Candida albicans, Saccharomyces cerevisiae*) and bacterial species has been used as a means to understand their interactions in the soil environment (84, 87, 88). Thus, a co-occurrence analysis between fungi and bacteria was performed to identify cross-kingdom interactions specific to the BRD and healthy group. In this study, the BRD animals had more positive cross-kingdom associations (bacterial and fungal ASVs that co-exist in the same sample) than the healthy animals. On the other hand, the healthy group had more negative cross-kingdom associations (bacterial and fungal ASVs that do not co-exist in the same sample) than the BRD group. These finding indicate that potentially these two kingdoms are more negatively correlated (inhibitive of other) in healthy animals. When investigating the cross-kingdom associated fungal ASVs, unfortunately, only three fungal ASVs associated with bacterial ASVs were identified at the genera level: *Alternaria, Cladosporium,* previously identified as “commensal” members of the nasal cavity (53, 59–63) and *Kluyveromyces,* which has been isolated from sputum samples from a patient with cystic fibrosis (89).

## Conclusion

Fungal ITS sequencing analysis demonstrated that at a taxonomy level, *Trichosporon* sp. and *Issatchenkia orientalis* were the most abundant species regardless of disease diagnosis and differential abundance at a sub-species level in these two species could be a potential indicator of BRD. No major difference in the fungal alpha and beta diversity was observed due to disease. However, changes in the fungal community structure were marked by seasonal effects, specifically when comparing samples collected in the summer to the winter season. Classification of BRD or healthy animals using ITS sequences agreed to some extent with the visual diagnosis and detected BRD animals than identified by the abundance of bacterial pathobionts. In addition, changes in specific members of the mycobiome community in the nasal cavity helped in the classification of disease status. Lastly, BRD animals had more fungi and bacterial co-occurrence than healthy animals. Nevertheless, is imperative to design studies that will increase the knowledge of how fungi and bacteria interact in the nasal cavity or any other part of the cattle respiratory tract to identify if fungi have a role in BRD development and any indicator that can help in the process of disease identification in addition to the bacterial and viral community composition and visualization of BRD clinical signs.

## Data availability statement

The original contributions presented in the study are publicly available. All the data and fungal statistical analysis were performed in R v.4.0.3 and the codes are available at https://github.com/EuniceCenteno/BRDFungal for reference and reproducibility. Fungal Sequences were deposited in the NCBI sequence read archive (SRA) database under Bioproject PRJNA906497, BioSamples SAMN31926246 - SAMN31926380.

## Ethics statement

The animal study was reviewed and approved by Purdue University Animal Care and Use Committee (protocol #1906001911). Written informed consent was obtained from the owners for the participation of their animals in this study.

## Author contributions

REC-M, MSV, and TAJ conceived the project. REC-M, SM, JD, JPS, and AA the acquired the nasal swab samples, REC-M analyzed the samples. REC-M and TAJ interpreted the data. REC-M, MSV, and TAJ wrote or substantively revised the manuscript. All authors contributed to the article and approved the submitted version.

## Funding

This work was supported by the Agriculture and Food Research Initiative Competitive Grants Program Award 2020-68014-31302 from the U.S. Department of Agriculture National Institute of Food and Agriculture. This work is also supported by Purdue University’s Colleges of Agriculture and Engineering Collaborative Projects Program 2018, and an Agricultural Science and Extension for Economic Development (AgSEED) grant. The funding organizations played no role in the design of the study: collection, analysis, and interpretation of data, or in writing the manuscript.

## Conflict of interest

MSV has interests in Krishi, Inc., a company interested commercializing on farm diagnostics technology. The work performed here was not funded by Krishi, Inc.

The remaining authors declare that the research was conducted in the absence of any commercial or financial relationships that could be construed as a potential conflict of interest.

## Publisher’s note

All claims expressed in this article are solely those of the authors and do not necessarily represent those of their affiliated organizations, or those of the publisher, the editors and the reviewers. Any product that may be evaluated in this article, or claim that may be made by its manufacturer, is not guaranteed or endorsed by the publisher.
