# Supplementary material for: The bovine nasal fungal community and associations with bovine respiratory disease
Source: Front Vet Sci. 2023 Jun 27;10:1165994. doi: 10.3389/fvets.2023.1165994 (PMC10335396; doi:10.3389/fvets.2023.1165994)
Supplement: Supplementary file 1 [file Data_Sheet_1.docx]

**SUPPLEMENTARY MATERIAL**

The bovine nasal fungal community and associations with bovine respiratory disease

Ruth Eunice Centeno-Martinez, Suraj Mohan, Josiah Levi Davidson, Jon Schoonmaker, Aaron Ault, Mohit S. Verma, Timothy A. Johnson

**Supplementary Figure S1.** PCR negative control (water) fungal community composition.

**Supplementary Figure S2.** The most influential fungal ASVs and BRD-pathobiont abundance present in the nasal cavity determined by random forest model to classify BRD or healthy animals. The 17 most influential fungal ASV in the nasal cavity determined by the mean decrease accuracy plotted in the x-axis **(A).** A total of 17 trees were set to build the fungal RF model based on the low error rate**.** BRD-pathobiont abundance importance to RF model **(B).**

**Supplementary Figure S3.** Phylogenetic tree of the genus *Issatchenkia* sp. identified based on the ITS gene-based sequencing data. The *Issatchenkia* ASV names represent the differentially abundance taxa enriched in the BRD or healthy animals. Phylogenetic tree was created using a ‘globalpair’ iterative refinement method with WSP and consistency score with 1,000 iterations.

**Supplementary Figure S4.** Phylogenetic tree of the genus *Trichosporon* sp. identified based on the ITS gene-based sequencing data. The *Trichosporon* ASV names represent the differentially abundance taxa enriched in the BRD or healthy animals. Phylogenetic tree was created using a ‘globalpair’ iterative refinement method with WSP and consistency score with 1,000 iterations. Black triangles indicate clades that were collapsed and contained other *Trichosporon* ASVs observed in the study but were not differentially abundance between the BRD and healthy animals.

**Table S1.** Fungi average relative abundance at phyla level in the nasal cavity of healthy (n=73) and BRD (n=56) cattle.

**Table S2.** Fungi average relative abundance at genus level in the nasal cavity of healthy (n=73) and BRD (n=56) cattle.


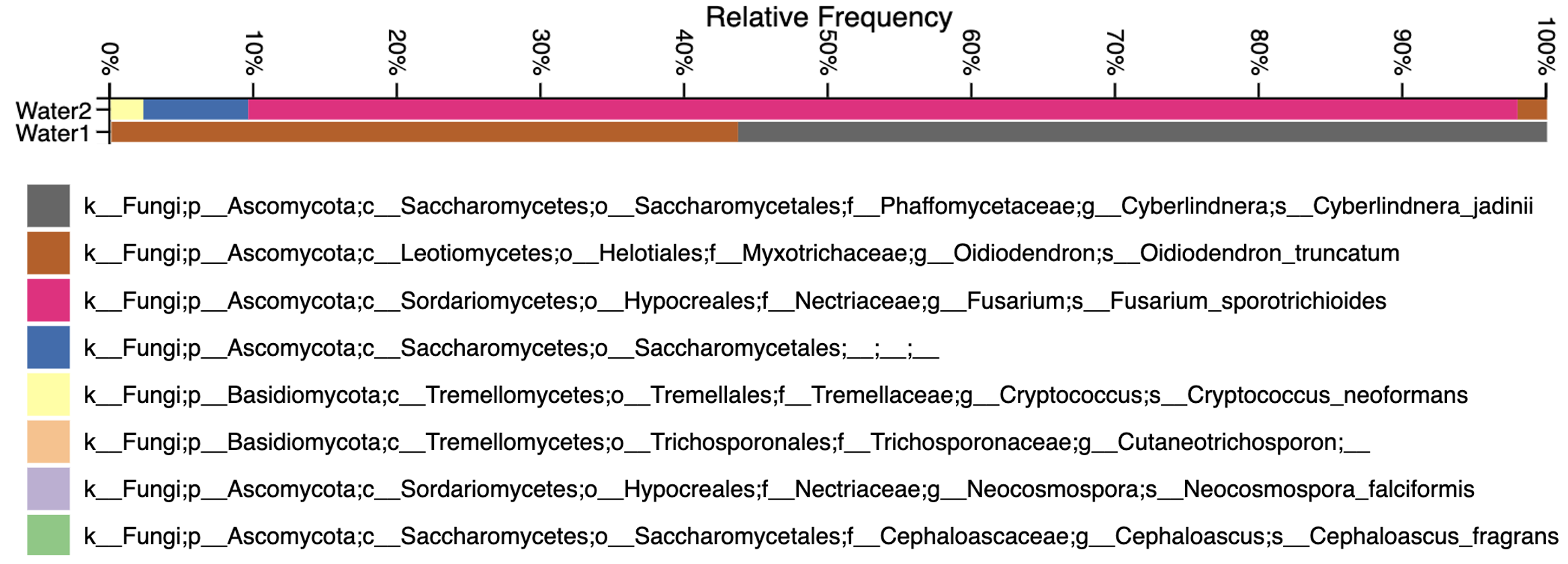


**Supplementary Figure S1.** PCR negative control (water) fungal community composition.


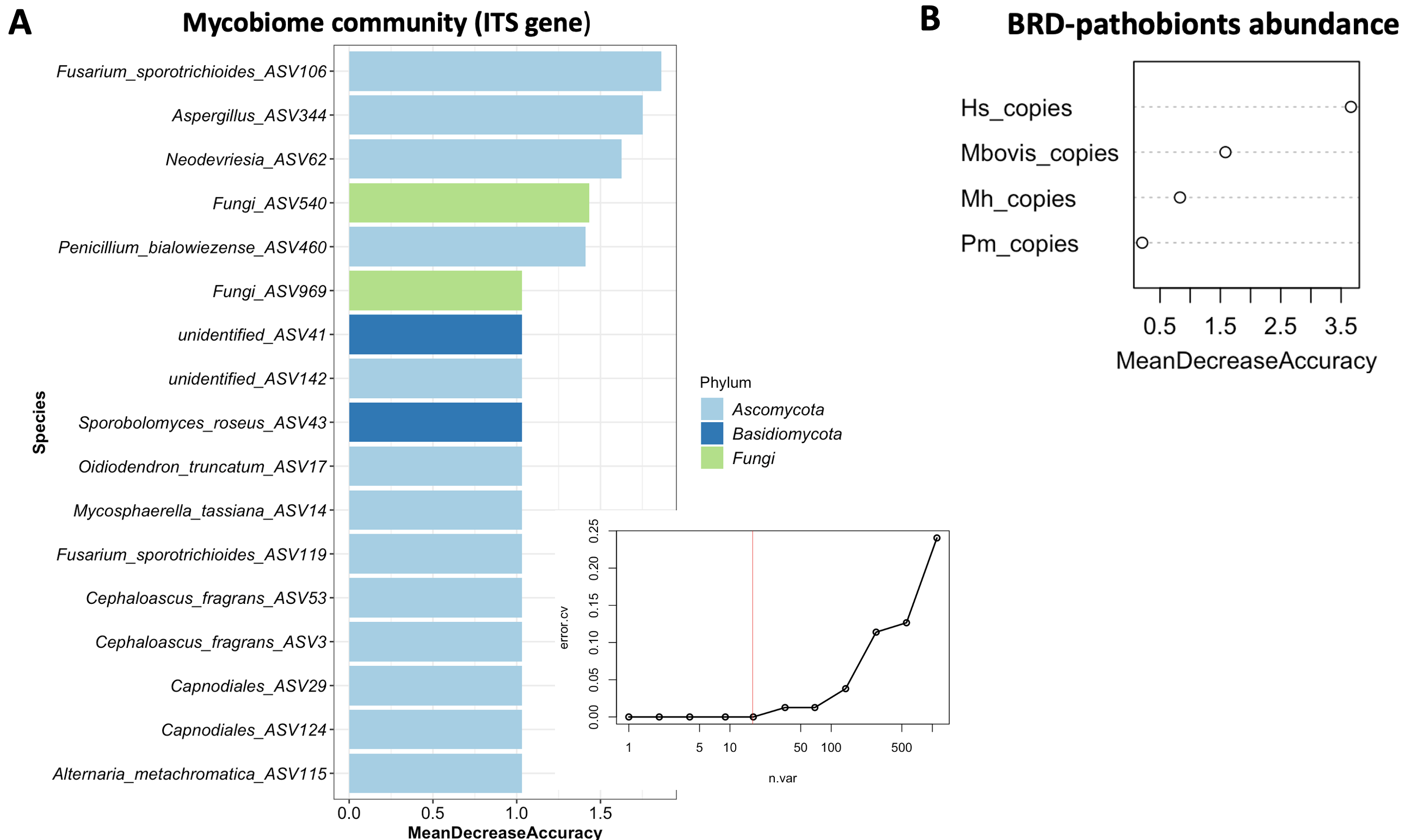


**Supplementary Figure S2.** The most influential fungal ASVs and BRD-pathobiont abundance present in the nasal cavity determined by random forest model to classify BRD or healthy animals. The 17 most influential fungal ASV in the nasal cavity determined by the mean decrease accuracy plotted in the x-axis **(A).** A total of 17 trees were set to build the fungal RF model based on the low error rate**.** BRD-pathobiont abundance importance to RF model **(B).**

*
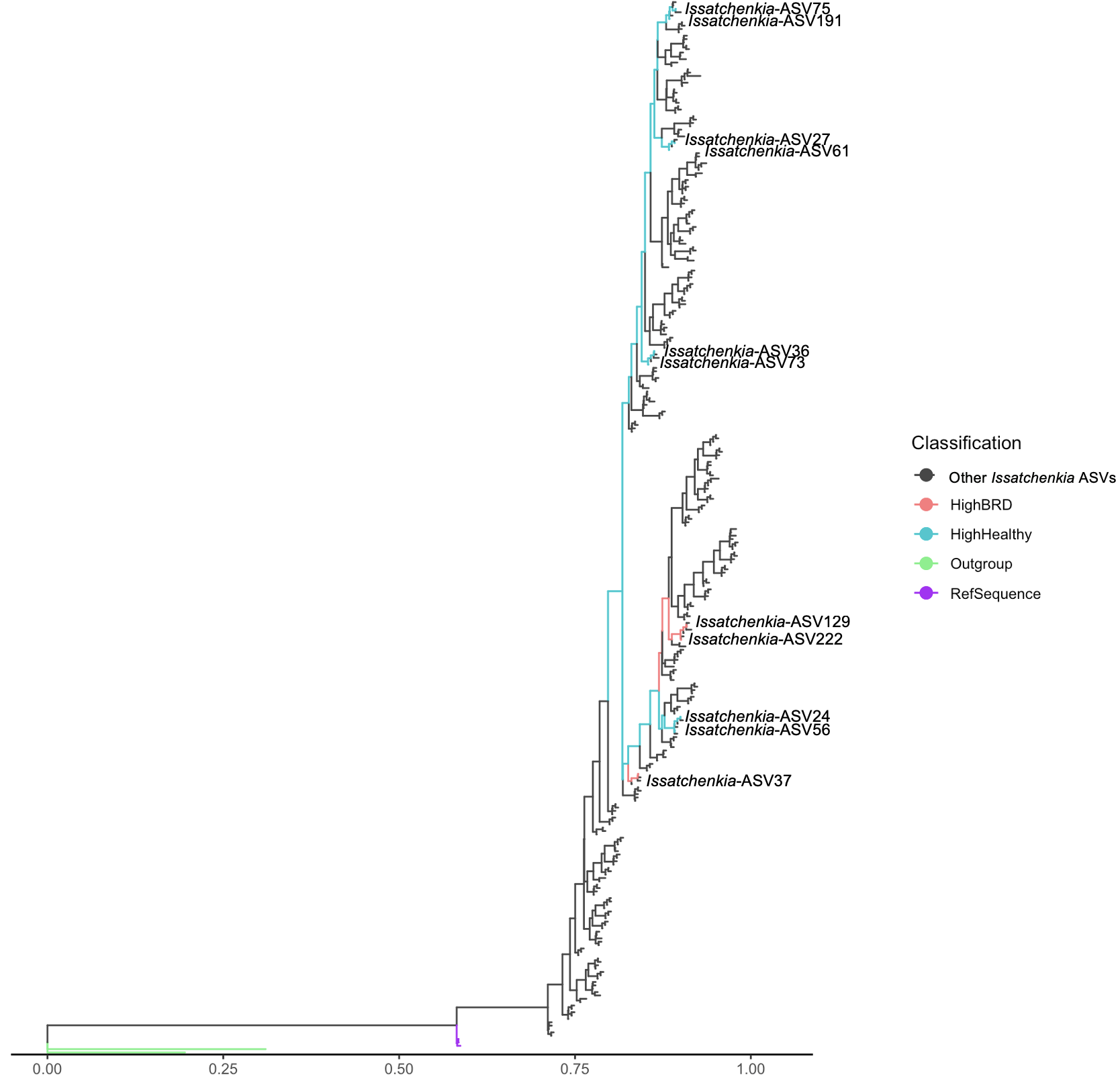
*

**Supplementary Figure S3.** Phylogenetic tree of the genus *Issatchenkia* sp. identified based on the ITS gene-based sequencing data. The *Issatchenkia* ASV names represent the differentially abundance taxa enriched in the BRD or healthy animals. Phylogenetic tree was created using a ‘globalpair’ iterative refinement method with WSP and consistency score with 1,000 iterations.

*
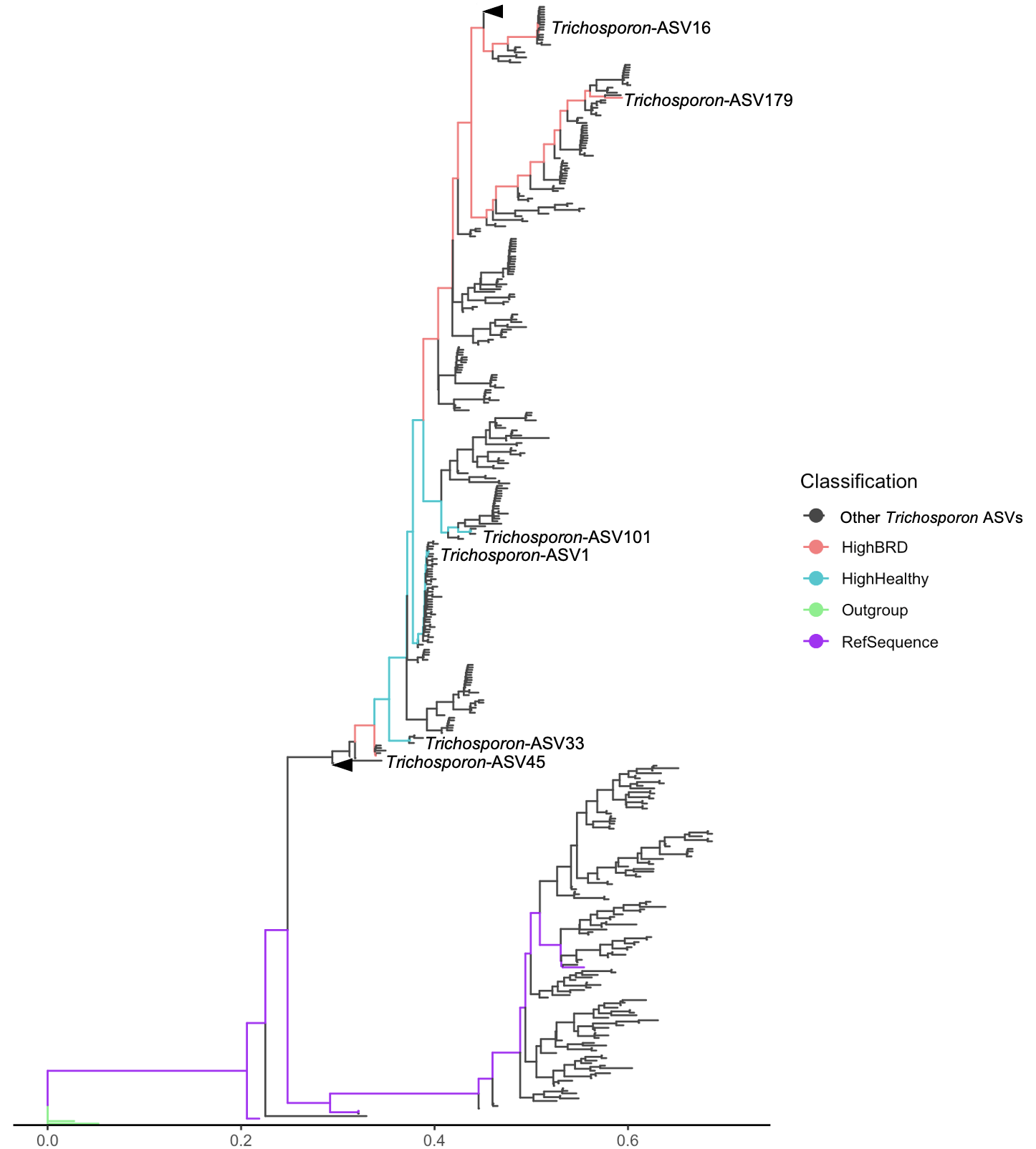
*

**Supplementary Figure S4.** Phylogenetic tree of the genus *Trichosporon* sp. identified based on the ITS gene-based sequencing data. The *Trichosporon* ASV names represent the differentially abundance taxa enriched in the BRD or healthy animals. Phylogenetic tree was created using a ‘globalpair’ iterative refinement method with WSP and consistency score with 1,000 iterations. Black triangles indicate clades that were collapsed and contained other *Trichosporon* ASVs observed in the study but were not differentially abundance between the BRD and healthy animals.
